# Comparative effectiveness of monotherapies and combination therapies for patients with hypertension: protocol for a systematic review with network meta-analyses

**DOI:** 10.1186/2046-4053-2-44

**Published:** 2013-06-28

**Authors:** Brian Hutton, Jennifer Tetzlaff, Fatemeh Yazdi, Justin Thielman, Salmaan Kanji, Dean Fergusson, Lise Bjerre, Edward Mills, Kristian Thorlund, Andrea Tricco, Sharon Straus, David Moher, Frans HH Leenen

**Affiliations:** 1 Ottawa Hospital Research Institute, 501 Smyth Road, Ottawa, ON, Canada, Box 201 K1H 8L6; 2 University of Ottawa Heart Institute, 40 Ruskin Street, Ottawa, ON, Canada K1Y 4W7; 3 Department of Family Medicine, University of Ottawa, 43 Bruyere Street (Floor 3JB), Ottawa, ON, Canada K1N 5C8; 4 Department of Epidemiology and Community Medicine, University of Ottawa, 451 Smyth Road, Ottawa, ON, Canada K1H 8M5; 5 Department of Clinical Epidemiology and Biostatistics, Faculty of Health Sciences, McMaster University, 1280 Main Street West, Hamilton, Ontario, Canada L8S 4 K1; 6 Li Ka Shing Knowledge Institute, St Michael’s Hospital, 209 Victoria Street, East Building, Toronto, ON, Canada M5B 1T8

**Keywords:** Hypertension, Pharmacotherapy, Systematic Review, Network Meta-analysis

## Abstract

**Background:**

Hypertension has been cited as the most common attributable risk factor for death worldwide, and in Canada more than one of every five adults had this diagnosis in 2007. In addition to different lifestyle modifications, such as diet and exercise, there exist many pharmaco-therapies from different drug classes which can be used to lower blood pressure, thereby reducing the risk of serious clinical outcomes. In moderate and severe cases, more than one agent may be used. The optimal mono- and combination therapies for mild hypertension and moderate/severe hypertension are unclear, and clinical guidelines provide different recommendations for first line therapy. The objective of this review is to explore the relative benefits and safety of different pharmacotherapies for management of non-diabetic patients with hypertension, whether of a mild or moderate to severe nature.

**Methods/Design:**

Searches involving MEDLINE and the Cochrane Database of Systematic Reviews will be used to identify related systematic reviews and relevant randomized trials. The outcomes of interest include myocardial infarction, stroke, incident diabetes, heart failure, overall and cardiovascular related death, and important side effects (cancers, depression, syncopal episodes/falls and sexual dysfunction). Randomized controlled trials will be sought. Two reviewers will independently screen relevant reviews, titles and abstracts resulting from the literature search, and also potentially relevant full-text articles in duplicate. Data will be abstracted and quality will be appraised by two team members independently. Conflicts at all levels of screening and abstraction will be resolved through team discussion. Random effect pairwise meta-analyses and network meta-analyses will be conducted where deemed appropriate. Analyses will be geared toward studying treatment of mild hypertension and moderate/severe hypertension separately.

**Discussion:**

Our systematic review results will assess the extent of currently available evidence for single agent and multi-agent pharmacotherapies in patients with mild, moderate and severe hypertension, and will provide a rigorous and updated synthesis of a range of important clinical outcomes for clinicians, decision makers and patients.

**Trial registration:**

PROSPERO Registration Number: CRD42013004459

## Background

Blood pressure (BP) is a measure of the pressure that blood places against the walls of blood vessels during circulation [1], measured in terms of systolic BP (SBP; normal values <130 mmHg) and diastolic BP (DBP; normal values <85 mmHg). When SBP rises above 140 mmHg or DBP rises above 90 mmHg, the patient is considered to have hypertension [1,2]. Hypertension is a chronic condition which places increased stress on the heart and blood vessels, and represents a critical risk factor for clinically significant events including myocardial infarction (MI), heart failure, stroke and death. Hypertension is the most common attributable risk factor for death worldwide, and an independent predictor of stroke mortality and ischemic heart disease mortality [3,4]. In 2007, more than one of every five adult Canadians suffered from high BP [5-7].

Reduction of elevated BP is associated with a reduction in the risk of clinically significant events. Evidence supports a direct correlation between the magnitude of BP reduction and the rate of major events, and thus treatment is geared toward lowering BP to below 140 SBP/90 DSP in most patients [8,9]. In addition to lifestyle changes, there exists a number of classes of antihypertensive pharmacotherapies which can be used to manage BP. Classes of agents include diuretics, angiotensin-converting enzyme (ACE) inhibitors, angiotensin receptor blockers, calcium channel blockers, beta blockers and alpha blockers, among others. Guidance from the Canadian Hypertension Education Program (CHEP) [2] suggests initial therapy should consist of monotherapy using any one among thiazide diuretics, beta-blockers, ACE inhibitors, long-acting calcium channel blockers or angiotensin receptor blockers (ARB). It is further recommended that use of a second agent should be considered if target BP is not achieved with monotherapy, with a choice made among the agents considered for first line monotherapy. More than 66% of patients with hypertension cannot have their BP adequately lowered using monotherapy, and so the addition of a second agent (and third) may be required [10].

A first question in clinical practice is about choice of therapy, and which treatment should be given to a patient first to minimize risk of undesirable outcomes. Pharmacotherapies are associated with different mechanisms of action, harm profiles and costs. The optimal choice of a first line agent remains unclear, as some works suggest that thiazide diuretics are best [11,12], while others suggest calcium channel blockers should be considered as first line therapy in patients over 55 years of age or of Caribbean or African descent [13]. In Canada, all but alpha blockers are considered reasonable first line therapies, with patients’ demographics and comorbidities playing a role in selection [2]. There also has been relatively little study to assess the relative effectiveness of combination therapies, which may be used in moderate to severe cases of hypertension.

To address these research gaps and discrepancies in guidance, the planned study will be a systematic review incorporating network meta-analyses to explore the relative effectiveness and safety of different monotherapies and combination therapies for hypertension. The planned review will include thiazide diuretics, ACE inhibitors, ARBs, calcium channel blockers, beta blockers, alpha blockers, placebo, no treatment and a combination antihypertensive therapy. Separate analyses to address benefits in different degrees of hypertension will be pursued.

## Methods/Design

### Selection of studies

The research question for this systematic review was specified according to the Population-Intervention-Comparator-Outcome-Study Design framework. The following criteria related to the study population, interventions and comparators, clinical outcomes and study designs of interest will be sought.

#### Population

Non-diabetic patients with hypertension are the target population of interest. No restrictions will be used regarding gender. Studies in adult patients will be retained, and subgroup analyses based on age (for example, age >60 years, age >75 years) will be explored. Studies of both first line therapy (that is, mainly monotherapies) and second line therapy (that is, mainly combination therapies) will be included. Studies related to hypertension during pregnancy, hypertensive emergencies and hypertension treatment in acute stroke will be excluded. Studies conducted in the following sub-populations will be eligible if all patients in the study were required to have hypertension upon entry, and if the benefits of BP lowering on outcomes were examined in the study: patients with micro- and macro-albuminuria; patients with metabolic disease; patients with myocardial ischemia; patients with coronary artery disease/atherosclerosis.

#### Interventions/Comparators

We will seek studies evaluating the following classes in monotherapy and combination therapy regimens: calcium channel blockers, beta blockers, angiotensin receptor blockers, alpha blockers, thiazide diuretics, potassium sparing diuretics and ACE inhibitors. Fixed doses for each agent will not be sought as an inclusion criterion, as it is anticipated that the majority of trials will have variable doses titrated for each patient.

#### Outcomes

Studies that report one or more of the following clinical outcomes will be included: MI, stroke, incident diabetes, overall and cardiovascular related death; adherence to treatment; and important adverse events which may impact quality of life (for example, sexual dysfunction, depression, syncopal episodes/falls, cancers). Where outcome definitions vary across studies, appropriate sensitivity analyses will be undertaken.

#### Study design

Randomized controlled trials of a minimum, one-year duration will be included in this review. Only English language studies will be retained, as past research suggests that limiting included studies to the English language does not greatly affect summary estimates from meta-analyses [14,15].

### Literature search strategy

Based on awareness of a large number of existing reviews and network meta-analyses that can be leveraged, an unlimited primary search for RCTs will not be performed. In its place, we will use a staged approach to study identification, beginning with the identification of relevant trials included in Cochrane systematic reviews searched for in the Cochrane Database of Systematic Reviews (publication years 2005 and onward) and existing network meta-analyses of which we are aware [16-21]. Medline (OVID interface) will next be searched to identify other relevant reviews, and we will systematically compare studies from relevant articles in reverse chronologic order until yield from this approach becomes low. The Medline search is presented in a supplemental Appendix, and was designed by a senior information specialist in consultation with the review team and peer reviewed using PRESS (Peer Review of Electronic Search Strategies) [22] by another experienced information specialist. To identify additional relevant RCTs published outside the time frames of these reviews, we will compile a list of the unique Medline identification numbers of all relevant articles, and perform a related articles search in Medline. Search results will be limited to the top general medicine journals and related specialty journals as determined by impact factor. This technique has been shown to be effective in identifying relevant studies [23], has been used recently by reviews in several fields including hypertension, and will increase efficiency in study identification in the presence of an already large and established evidence base.

### Study selection and data extraction

Review of citations based on title, keywords and abstract (Level 1 screening) and full text articles (Level 2 screening) will be carried out independently by two reviewers. Level 1 citations deemed potentially relevant or unclear will be carried forward to Level 2. Study selection will be conducted using Distiller Systematic Review Software (DSR; Ottawa, ON, Canada). Where consensus is not achieved following discussion, a third member of the research team will be consulted to settle disagreements. The process of literature selection will be reported using a flow diagram as recommended by the PRISMA (Preferred Reporting Items for Systematic Reviews and Meta-Analyses) statement [24].

### Data extraction and risk of bias assessment

Primary data collection will be performed independently by two reviewers using a standardized data collection form implemented using SR Distiller Software (Evidence Partners Incorporated, Ottawa, Ontario, Canada); this software will also be used to compare collected data for accuracy and agreement, with disagreements being settled by discussion. The following information will be collected from all eligible studies: authorship list, year and journal of publication, countries of study, funding source, group sample sizes, study inclusion criteria, age distribution, gender distribution, ethnicity distribution, patient comorbidity history, past/present medication use and all relevant outcome information. To assess study risk of bias, all RCTs will be reviewed using the Cochrane Risk of Bias (RoB) tool [25]. Disagreements will be resolved through discussion or by third party adjudication.

### Data analysis

Studies of first line therapy (that is, mainly monotherapies unless otherwise rationalized to consider combination therapies) and second line therapy (that is, mainly combination therapies) will be analyzed separately to maintain clinical homogeneity in terms of patient characteristics. Among combination therapy regimens, we will also explore analyses to assess whether there is a differential clinical benefit between combinations involving a thiazide diuretic versus those which do not.

#### Approach to analysis

We will begin with a narrative overview of studies included in the review which will provide insights regarding the degree of clinical and methodologic homogeneity among included RCTs, thereby helping to explore the assumptions of homogeneity and similarity for network meta-analysis [26]. Study of statistical heterogeneity as measured by values of I^2^ within pairwise meta-analyses will also contribute to this assessment. Where assumptions are judged reasonable, network meta-analysis (NMA) will be carried out for each clinical outcome separately; NMA is an approach to evidence synthesis which allows for the combination of direct and indirect evidence to compare three or more treatments in a unified analysis. Indirect comparisons between treatments A and B based on a common comparator C where no trials of A versus B exist (that is, no direct evidence) but trials of A versus C and B versus C exist (that is, indirect evidence) were originally proposed by Bucher *et al.*[27], and Lumley [28] and Lu and Ades [29] subsequently developed extensions of this methodology. In addition to estimating all pairwise comparisons between treatments in a network, this technique can also be used to estimate probabilities of treatment of superiority to rank the treatments. As part of this will involve analyses that include combination therapies, recent methods proposed by Mills and Thorlund may be explored [30].

#### Addressing clinical and methodological heterogeneity

Findings from risk of bias assessments of included studies will inform sensitivity analyses, including meta-regression or exclusion of higher risk studies to address the impact of perceived study deficiencies. Meta-regression and/or removal of studies from the treatment network to address clinically important variations between studies with regard to gender distribution, age distribution, history of chosen clinically relevant baseline comorbidities and other relevant factors will be considered. If several trials contain percentages of diabetic patients, sensitivity analyses excluding these studies will be performed.

Clinical practice for patients with hypertension has evolved over time, including the emergence of common use of co-interventions such as statins and aspirin. To explore the potential impact of such changes, we will explore meta-regression analyses using the network meta-analysis approach to adjust for year of initiation of patient enrollment in each study. This may be a helpful proxy in the absence of other information about background treatments.

#### Assessment of coherence

This review will consist of a mixture of both direct evidence (that is, head to head comparisons between different antihypertensive agents or classes) and indirect evidence (that is, inactively controlled studies of the different agents or classes). As described elsewhere [31,32], there is a need to verify that findings generated from synthesis of direct and indirect data do not differ more than one would expect by chance. We will employ methods described by Dias *et al.*[33] to assess the validity of this assumption in this work. As statistics alone cannot always be replied upon to identify important clinical or methodologic differences between studies, we will also employ evidence tables of study characteristics to further assess homogeneity.

#### Reporting of findings from analyses

Full graphical and numeric presentations of findings [34] will be provided to convey the results of our work. This will include network diagrams showing the structure of available evidence for all possible treatment comparisons, summary point estimates and 95% credible intervals for all pairwise comparisons, and estimated probabilities that each therapy is deemed ‘best’ for each outcome along with associated average rankings. Forest plots of summary estimates as well as rankograms [34] will be used to clearly delineate any important variations from the primary analysis for subgroup effects.

## Discussion

The proposed review will add to the literature in several ways. To our knowledge, the planned review includes analyses for certain clinical outcomes which have not been the subject of past network meta-analyses, and others which are important for consideration of harms and patients’ quality of life. The review will also pursue updates of comparisons of therapies using a network meta-analysis approach for several clinical outcomes which have not been studied using this technique since 2003, and thus for which considerable new data are likely available. The review will generate new information by addressing combination therapies for hypertension, both (i) in comparison to monotherapies in specific patient populations where either might be used, and (ii) in patients who have moderate to severe hypertension. To our knowledge, neither has been addressed using network meta-analysis. Finally, we will also improve upon limitations of past reviews by including improved assessment and accounting for the effects of clinical heterogeneity between studies.

There are potential challenges to the planned review. Clinical expertise and preliminary review of a sample of relevant trials for this research shows that in some studies, while patients are randomized to one active antihypertensive agent to address the study’s research question, additional anti-hypertensive medications may be prescribed to patients at the physicians’ discretion. While these additional treatments may have implications for the additional effects seen, outcome data for those remaining strictly on the prescribed treatments may not be available. This may be a limiting factor concerning the ‘purity’ of some included trial data. We anticipate at least a moderate degree of clinical heterogeneity with regard to the clinical populations enrolled from study to study. If the extent of included literature turns out to be small, the ability to explore heterogeneity could be limited; however, past works suggest considerable evidence will be found. There are many possible outcomes of interest to be studied in relation to hypertensive patients. We have attempted to identify outcomes that are clinically important and can be studied using network meta-analysis to produce important information in a timely fashion.

## Appendix

### **Appendix: Medline search strategy**

Database: Ovid MEDLINE(R) In-Process and Other Non-Indexed Citations and Ovid MEDLINE(R) <1946 to Present> Search Strategy:

1. exp Hypertension/ (199996)

2. hypertens*. tw. (294265)

3. ((high* or rais* or elevat* or heighten* or increas*) adj3 (“blood pressure” or “diastolic pressure” or “systolic pressure” or “pulse pressure”)). tw. (46697)

4. ((high* or rais* or elevat* or heighten* or increas*) adj3 (BP or DBP or SBP)). tw. (11512)

5. exp Cardiovascular Diseases/pc [Prevention & Control] (142328)

6. ((borderline or pre-disease* or pre-clinical* or preclinical* or sub-clinical* or subclinical* or pre-morbid* or premorbid* or risk* or susceptib* or pre-dispos* or predispos* or predict* or probabilit* or likelihood or likeliness or prevent*) adj3 (cardiovascular or cardiometabolic* or cardio-metabolic* or coronary disease* or heart disease* or heart attack* or heart failure or myocardial infarction* or coronary artery disease* or CVD or peripheral artery disease* or PAD or CHD or CAD or arteriosclerosis or atherosclerosis or stroke)). tw. (130079)

7. or/1-6 (568110)

8. exp hypertension/dt [drug therapy] (54046)

9. exp Sodium Chloride Symporter Inhibitors/ (11688)

10. ((thiazide or benzothiadiazine or benzo-thiadiazine or potassium depleting) adj1 diuretic$1).tw. (2092)

11. (sodium chloride symporter inhibitor$1 or sodium chloride cotransporter inhibitor$1 or sodium chloride co-transporter inhibitor$1 or thiazide sensitive NaCl cotransporter inhibitor$1 or thiazide sensitive NaCl co-transporter inhibitor$1). tw. (0)

12. exp Chlorothiazide/ (7612)

13. (chlorothiazide or Alurene or Chlorosal or Chlotride or Chlorothiazid or Chlorothiazidum or Chlorthiazid or Chlorthiazide or Chrthiazidum or Chlortiazid or Chlorurit or Chlotride or Clorotiazide or Clotride or Diuresal or Diuril or Diurilix or Diurite or Diutrid or Flumen or Minzil or Neo-dema or SK-Chlorothiazide or Salisan or Salunil or Saluretil or Saluric or Thiazide or Urinex or Warduzide or Yadalan). tw. (4691)

14. 58-94-6.rn. (2058)

15. exp Chlorthalidone/ (1316)

16. (chlorthalidone or Apo-Chlorthalidone or Chlorphthalidolone or Chlorphthalidone or Chlortalidone or Chlortalidonum or Chlorthalidon or Clortalidone or Famolin or Hydro-Long or Hygroton or Igroton or Isoren or Natriuran or Oksodolin or oxodolin or Oradil or Oxodolin or Phthalamodine or Phthalamudine or Racemic chlorthalidone or Renon or Saluretin or Thalitone or Urolin or Zambesil). tw. (1144)

17. 77-36-1.rn. (1316)

18. exp Hydrochlorothiazide/ (5737)

19. (Hydrochlorothiazide or “Aquazide H” or Apo-Hydro or Carozide or Dichlothiazide or Dihydrochlorothiazide or Esidrex or Esidrix or Ezide or HCTZ or Hydrochlorot or HydroDIURIL or Hydro-par or HydroSaluric or Hypothiazide or Microzide or Oretic or Sectrazide). tw. (5452)

20. 58-93-5. rn. (5554)

21. exp Hydroflumethiazide/ (141)

22. (Hydroflumethiazide or Bristab or Bristurin or Di-ademil or Di-adenil or Dihydroflumethiazide or Diucardin or Diuredemina or Diurometon or Elodrin or Elodrine or Enjit or Finuret or Flutizide or Hydol Hydrenox or Hydroflumethiazide or Hydroflumethiazidum or Hydroflumethizide or Idroflumetiazide or Leodrine or NaClex or Olmagran or Rivosil or Robezon or Rodiuran or Rontyl or saluron or Sisuril or Spandiuril or Trifluoromethylhydrothiazide or Vergonil).tw. (128)

23. 135-09-1. rn. (141)

24. exp Indapamide/ (850)

25. (indapamide or Arifon or Bajaten or Cormil or Damide or Fludex or Indaflex or Indapamide or Indamol or Ipamix or Lozol or Metindamide or Natrilix or Noranat or Pressurai or Tandix or Tertensif or Veroxil). tw. (952)

26. 26807-65-8.rn. (850)

27. exp Methyclothiazide/ (106)

28. (methyclothiazide or Aquatensen or Enduron or Naturon). tw. (105)

29. 135-07-9. rn. (106)

30. exp Metolazone/ (156)

31. (metolazone or Diulo or Microx or Mykrox or Oldren or Zaroxolyn or Zytanix). tw. (241)

32. 17560-51-9. rn. (156)

33. exp Polythiazide/ (237)

34. (Polythiazide or Drenusil or Nephril or Polythiazidum or Renese). tw. (198)

35. 346-18-9. rn. (237)

36. exp Angiotensin-Converting Enzyme Inhibitors/ (38358)

37. ((Angiotensin-Converting Enzyme or Angiotensin I-Converting Enzyme or ACE or Kininase II) adj (inhibitor* or antagonist*)). tw. (26527)

38. (ACEI or ACEIs). tw. (2465)

39. exp Captopril/ (9515)

40. (Acediur or Aceplus or Acepress or Acepril or Alopresin or Asisten or Capoten or Captolane or Captopril or Captoprilum or Captopryl or Captoril or Cesplon or Dilabar or Farcopril or Garranil or Hypertil or Hypopress or Isopresol or “L-Captopril” or Lopirin or Lopril or Novocaptopril or Tenosbon or Tensoprel or Zapto). tw. (10387)

41. 62571-86-2.rn. (9515)

42. exp Enalapril/ (6105)

43. (Enalapril or Bonuten or Enalaprila or Enalaprilum or Gadopril or Kinfil). tw. (5429)

44. 75847-73-3. rn. (5546)

45. exp Lisinopril/ (1766)

46. (Lisinopril or Lisinopril dehydrate or Prinivil or Renacor or Zestril). tw. (1989)

47. 83915-83-7.rn. (1766)

48. (Benazepril hydrochloride or Benazepril HCl or Briem or Cibace or Cibacen or Cibacen CHF or Cibacene or Labopol or Lotensin or Lotrel or Tensanil or Zinadril). tw. (95)

49. benazepril. rn. (467)

50. exp Fosinopril/ (386)

51. (Fosinopril or Dynacil or Fosenopril or Fosinil or Fosinorm or Fositens or Fozitec or Hiperlex or Monopril or Newace or Staril or “Tenso Stop” or Tensocardil). tw. (462)

52. 98048-97-6. rn. (386)

53. exp Ramipril/ (1740)

54. (Ramipril or Acovil or Altace or Carasel or Cardace or Delix or Hytren or Lostapres or Naprix or Pramace or Quark or Ramace or Ramiprilum or Ramipro or Triatec or Tritace or Vesdil or Zabien).tw. (2455)

55. 87333-19-5. rn. (1740)

56. (Quinapril hydrochloride or Accupril or Accuprin or Accupron or Acequin or Acuitel or Acuprel or Asig or Conan or Continucor or Ectren or Hemokvin or Korec or Koretic or Lidaltrin or Quinapril or Quinapril HCl or Quinazil). tw. (805)

57. 82586-55-8. rn. (608)

58. exp Perindopril/ (1389)

59. (Aceon or Covapril or Coversyl or Perindopril or Pirindopril or Prestarium). tw. (1470)

60. 82834-16-0. rn. (1389)

61. (Trandolapril or Gopten or Mavik or Odrik or Udrik). tw. (569)

62. 87679-37-6.rn. (483)

63. (Moexiril or Fempress or Moex or Moexipril hydrochloride or Perdix or Univasc). tw. (70)

64. 103775-10-6. rn. (74)

65. exp Calcium Channel Blockers/ (69679)

66. ((calcium or ca) adj2 (blocker* or blockader* or blocking or antagonist* or inhibitor*)). tw. (33221)

67. exp Amlodipine/ (2732)

68. (Amlodipine or Amlodipine Besylate or Amlodipine Maleate or Amlodis or Amlor or Astudal or Coroval or Istin or Lipinox or Norvasc).tw. (3298)

69. 88150-42-9. rn. (2732)

70. (Aranidipine or Sapresta). tw. (14)

71. 86780-90-7. rn. (27)

72. (Azelnidipine or Calblock). tw. (148)

73. 123524-52-7. rn. (125)

74. (Barnidipine or Cyress or HypoCa or Libradin or Mepirodipine). tw. (78)

75. 104713-75-9. rn. (59)

76. (Benidipine or Benidipinum or Coniel). tw. (254)

77. 105979-17-7. rn. (185)

78. (Cilnidipine or Atelec or Cinalong or Siscard). tw. (164)

79. 132203-70-4. rn. (132)

80. (Clevidipine or Cleviprex). tw. (89)

81. clevidipine. rn. (68)

82. exp Isradipine/ (1325)

83. (Isradipine or Dynacirc or “DynaCirc CR” or Isradipinum or Lomir or Prescal). tw. (1079)

84. 75695-93-1. rn. (1325)

85. (Efonidipine or Landel). tw. (176)

86. efonidipine.rn. (109)

87. exp Felodipine/ (1083)

88. (Felodipine or Agon or Felo Biochemie or Felo-Puren or Felobeta or Felocor or Felodipin or Felodur or Felogamma or Fensel or Flodil or Modip or Munobal or Perfudal or Plendil or Renedil). tw. (1370)

89. 72509-76-3. rn. (1083)

90. (Lacidipine or Lacidipinum or Lacimen or Lacipil or Motens). tw. (372)

91. 103890-78-4.rn. (286)

92. (Lercanidipine or Lacidipinum or Lercadip or Lerdip or Zanidip). tw. (184)

93. 103890-78-4. rn. (286)

94. (Manidipine or Calslot or Madipine or Franidipine). tw. (226)

95. 89226-50-6. rn. (181)

96. exp Nicardipine/ (2364)

97. (Nicardipine or Antagonil or Carden SR or Cardene or Dagan or Flusemide or Lecibral or Lincil or Loxen or Lucenfal or Nicardipinum or Perdipine or Ridene or Vasonase). tw. (3363)

98. 55985-32-5. rn. (2364)

99. exp Nifedipine/ (14811)

100. (Nifedipine or Adalat or Afeditab or Citilat or Cordipin or Cordipine or Corinfar or Fenihidin or Fenihidine or Fenigidin or Korinfar or Nifediac or Nifedical or Nifangin or Oxcord or Procardia or Procardia XL or Vascard). tw. (17914)

101. 21829-25-4.rn. (14811)

102. (Nilvadipine or Escor or Nivadil or Nilvadipinum). tw. (272)

103. 75530-68-6. rn. (238)

104. exp Nimodipine/ (2372)

105. (Nimodipine or Admon or Brainal or Calnit or Kenesil or Modus or Nimodipin or Nimodipinum or Nimotop or Periplum or Remontal). tw. (3996)

106. 66085-59-4. rn. (2372)

107. exp Nisoldipine/ (734)

108. (Nisoldipine or Baymycard or Nisocor or Nisoldipinum or Sular or Syscor). tw. (973)

109. 63675-72-9. rn. (734)

110. 110.exp Nitrendipine/ (2061)

111. (Nitrendipine or Balminil or Bayotensin or Baylotensin or Baypresol or Baypress or Cardif or Gericin or Jutapress or Nidrel or Niprina or Nitre AbZ or Nitre-Puren or Nitregamma or Nitren 1A Pharma or Nitren acis or Nitren Lich or Nitrend KSK or Nitrendepat or Nitrendi Biochemie or Nitrendidoc or Nitrendimerck or Nitrepin or Nitrendipin or Nitrendipino or Nitrensal or Nitrepress or Nitrendipinum or Tensogradal or Trendinol or Vastensium). tw. (2755)

112. 39562-70-4. rn. (2061)

113. (Pranidipine or Acalas). tw. (33)

114. 99522-79-9. rn. (36)

115. exp Verapamil/ (16518)

116. (Verapamil or Calan or Cordilox or Dexverapamil or Dilacoran or Falicard or Finoptin or Iproveratril or Isoptimo or Isoptin or Isoptine or Izoptin or Lekoptin or Vasolan or Verapamilum or dl-Verapamil). tw. (20352)

117. 52-53-9. rn. (15706)

118. exp Diltiazem/ (5862)

119. (Diltiazem or Aldizem or Cardil or Cardizem or Cardizem LA or Dilacor or Dilacor XR or Dilcontin or Dilren or Dilta-Hexal or Diltiazem Hydrochloride or Diltiazem Malate or Diltiazemum or Dilticard or Dilzem or Endrydil or Incoril AP or Tiazac). tw. (7899)

120. 42399-41-7. rn. (5862)

121. exp Mibefradil/ (519)

122. (mibefradil or Posicor). tw. (621)

123. 116644-53-2. rn. (519)

124. exp Bepridil/ (701)

125. (Bepridil or Bedapin or Bepadin or Cordium or Unicordium or Vascor). tw. (698)

126. 64706-54-3. rn. (701)

127. exp Fluspirilene/ (103)

128. (fluspirilene or Fluspi or Fluspirilenum or Imap or Kivat or Redeptin). tw. (226)

129. 1841-19-6. rn. (103)

130. exp Aldosterone Antagonists/ (7103)

131. (aldosterone adj (antagonist* or inhibit*)). tw. (1112)

132. exp Spironolactone/ (5381)

133. (spironolactone or Acelat or Aldace or Aldactone or Alderon or Aldopur or Almatol or Altex or Aquareduct or Berlactone or duraspiron or Diatense or Espironolactona or Euteberol or Flumach or Frumikal or Jenaspiron or Novo-Spiroton or Practon or Spiractin or Spiresis or Spirobeta or Spirogamma or Spirolactone or Spirolang or Spirono-Isis or Spironone or Spirospare or Uractone or Urusonin or Veroshpiron or Verospiron or Verospirone or Xenalon). tw. (4652)

134. 52-01-7. rn. (5381)

135. (Eplerenone or Inspra). tw. (684)

136. Eplerenone. rn. (541)

137. exp Adrenergic Antagonists/ (111121)

138. ((Adrenergic or alpha-adrenergic or beta-adrenergic) adj3 (block* or alpha-block* or beta-block* or antagonist* or alpha-antagonist* or beta-antagonist*)). tw. (17756)

139. ((alpha1 or “alpha-1” or alpha2 or “alpha-2” or beta or beta1 or “beta-1” or beta2 or “beta-2” or beta3 or “beta-3”) adj2 (block* or antagonist*)). tw. (54717)

140. (adrenolytic* or anti-adrenergic* or antiadrenergic*). tw. (1347)

141. exp Phenoxybenzamine/ (4990)

142. (Phenoxybenzamine or Bensylyt or Benzylyt or Dibenylene or Dibenyline or Dibenziran or Dibenzylin or Dibenzyline or Dibenzyran or Fenossibenzamina or Fenoxibenzamina or Phenoxybenzaminum). tw. (3753)

143. 59-96-1. rn. (4990)

144. exp Phentolamine/ (9050)

145. (Phentolamine or Dibasin or Fentolamin or Phentolaminum or Regitine or Regityn or Rogitine or “Z-Max”). tw. (9471)

146. 50-60-2. rn. (9050)

147. exp Tolazoline/ (993)

148. (Tolazoline or Artonil or Benzalolin or Benzazoline or Benzidazol or Benzolin or Benzylimidazoline or Dilatol ASI or Divascol or Imidalin or Kasimid or Lambril or Olitensol or Peripherine or Phenylmethylimidazoline or Prefaxil or Pridazole or Priscol or Priscoline or Tolazolin or Tolazolinum or Vasimid or Vasodil or Vasodilatan).tw. (874)

149. 59-98-3. rn. (993)

150. (Alfuzosin or Alfetim or Afusozine or Alphuzosine or Alfuzosinum or Benestan or Urion or UroXatral or Xatral). tw. (383)

151. 81403-80-7. rn. (307)

152. exp Prazosin/ (8129)

153. (Prazosin or Furazosin or Minipress or Pratsiol or Prazosinum). tw. (9945)

154. 19216-56-9. rn. (7348)

155. exp Doxazosin/ (1097)

156. (Doxazosin or Alfamedin or Apo-Doxazosin or Cardular or Cardura or Carduran or Carduran Neo or Diblocin or Doxa-Puren or Doxacor or Doxagamma or Doxamax or Doxatensa or DoxaUro or Doxazomerck or Doxazosine or Doxazosinum or Gen-Doxazosin or Jutalar or MTW-Doxazosin or Novo-Doxazosin or Progandol Neo or Uriduct or Zoxan). tw. (1257)

157. 74191-85-8. rn. (1097)

158. (Tamsulosin or Flomax or Tamsulosine or Tamsulosinum). tw. (973)

159. 106133-20-4. rn. (769)

160. (Terazosin or Adecur or Apo-Terazosin or Blavin or Deflox or Dysalfa or Flotrin or Flumarc or Fosfomic or Heitrin or Hytrin or Hytrine or Magnurol or Novo-Terazosin or Nu-Terazosin or Sutif or Tazusin or Terazoflo or Vasomet or Zayasel). tw. (691)

161. 63590-64-7. rn. (529)

162. (Atipamezole or Antisedan or Atipamezol or Atipamezolum). tw. (597)

163. 104054-27-5. rn. (405)

164. exp Idazoxan/ (1388)

165. (Idazoxan or Idazoxanum). tw. (1828)

166. 79944-58-4. rn. (1388)

167. exp Yohimbine/ (19692)

168. (Yohimbine or Aphrosol or Aphrodine or Aphrodyne or Corynine or Corynanthine or Pluriviron or Quebrachin or Quebrachine or Rauhimbine or Rauwolscine or Yocon or Yohimbin or Yohimex). tw. (7878)

169. 146-48-5. rn. (5437)

170. (Carvedilol or Carvedilolum or Coreg or Coropres or Dilatrend or Eucardic or Kredex or Querto). tw. (2195)

171. 72956-09-3. rn. (1965)

172. exp Labetalol/ (1667)

173. (Labetalol or Albetol or Apo-Labetalol or Dilevalol or Dilevalolum or Labetolol or Normodyne or Presolol or Trandate). tw. (1897)

174. 36894-69-6. rn. (1667)

175. exp Alprenolol/ (2174)

176. (Alprenolol or Alfeprol or Alpheprol or Alprenololum or Aptin or Aptin-Duriles or Aptina or Aptine) .tw. (983)

177. 13655-52-2. rn. (1067)

178. (Bucindolol or Bucindololum). tw. (138)

179. Bucindolol. rn. (110)

180. exp Carteolol/ (336)

181. (Carteolol or Carteololum). tw. (425)

182. 51781-06-7. rn. (336)

183. exp Nadolol/ (750)

184. (Nadolol or Anabet or Corgard or Corzide or Nadololum or Solgol). tw. (1031)

185. 42200-33-9. rn. (750)

186. exp Oxprenolol/ (1011)

187. (Oxprenolol or Coretal or Koretal or Oxprenololum or Slow Trasicor or Tevacor or Trasicor). tw. (1036)

188. 6452-71-7. rn. (1011)

189. exp Penbutolol/ (174)

190. (Penbutolol or Betapressin). tw. (236)

191. 36507-48-9. rn. (174)

192. exp Pindolol/ (3689)

193. (Pindolol or Betapindol or “Blocklin L” or Calvisken or Carvisken or Decreten or Durapindol or Glauco-Viskin or Pectobloc or Pinbetol or Pindololum or Prinodolol or Pyn astin or Visken).tw. (2706)

194. 13523-86-9. rn. (3614)

195. exp Propranolol/ (30773)

196. (Propranolol or Anaprilin or Anapriline or Avlocardyl or Betadren or Betalong or beta-Propranolol or Corpendol or Dexpropranolol or Dociton or Euprovasin or Inderal or Obsidan or Obzidan or Propanix or Propranololum or Reducor or Sawatal or Sumial or Rexigen). tw. (30160)

197. 525-66-6. rn. (30773)

198. exp Sotalol/ (1903)

199. (Sotalol or Darob or Sotalolum or beta-Cardone).tw. (2376)

200. 3930-20-9. rn. (1903)

201. exp Timolol/ (3166)

202. (Timolol or Blocadren or Timacar). tw. (3532)

203. 26839-75-8. rn. (3166)

204. Eucommia bark$1. tw. (7)

205. exp Atenolol/ (4674)

206. (Atenolol or Tenormin or Tenormine). tw. (6104)

207. 29122-68-7. rn. (4674)

208. exp Betaxolol/ (624)

209. (Betaxolol or Betaxololum). tw. (783)

210. 63659-18-7. rn. (624)

211. exp Bisoprolol/ (771)

212. (Bisoprolol or Concor). tw. (956)

213. 66722-44-9. rn. (771)

214. exp Celiprolol/ (380)

215. (Celiprolol or Celiprololum or Selectol). tw. (467)

216. 56980-93-9. rn. (380)

217. Esmolol. tw. (935)

218. Esmolol. rn. (768)

219. exp Metoprolol/ (4611)

220. (Metoprolol or Beatrolol or Beloc-Duriles or Betaloc or Betalok or Corvitol or Lopressor or Meijoprolol or Metohexal or Metoprololum or Metrol or Minax or Neobloc or Preblok or Presolol or Selokeen or Seloken or Spesicor or Spesikor or Toprol). tw. (5533)

221. 37350-58-6. rn. (4611)

222. (Nebivolol or Bystolic or Lobivon or Nebilet or Nobiten or Silostar or Vasoxen). tw. (603)

223. Nebivolol. rn. (534)

224. exp Angiotensin Receptor Antagonists/ (15906)

225. (angiotensin adj3 (antagonist* or block*)). tw. (13473)

226. (Sartan or Sartans). tw. (154)

227. ARBS. tw. (1784)

228. exp Losartan/ (5446)

229. (Losartan or Cozaar or Losartan Monopotassium Salt or Losartan Potassium). tw. (6460)

230. 114798-26-4. rn. (5446)

231. Candesartan. tw. (1988)

232. 139481-59-7. rn. (1526)

233. (Valsartan or Diovan or Kalpress or Miten or Nisis or Provas or Tareg or Vals or Valtan or Valzaar). tw. (1957)

234. 137862-53-4. rn. (1522)

235. (Irbesartan or Aprovel or Avapro or Karvea). tw. (1154)

236. 138402-11-6. rn. (948)

237. (Telmisartan or Kinzalmono or Micardis or Pritor). tw. (1220)

238. 144701-48-4. rn. (1023)

239. (Eprosartan or Teveten). tw. (272)

240. 133040-01-4. rn. (244)

241. (Benicar or Olmesartan or Omesartan or Olmetec or Votum). tw. (796)

242. 144689-24-7. rn. (293)

243. Azilsartan. tw. (28)

244. Azilsartan. rn. (17)

245. exp Hydralazine/ (4220)

246. (Hydralazine or Apresoline or Apressin or Apressoline or Aprezolin or Hydrallazin or Hydrazinophthalazine or Nepresol). tw. (3916)

247. 86-54-4. rn. (4005)

248. (Dihydralazine or Depressan or Dihydrallazine or Dihydrazinophthalazin or Hypopresol or Nepresol or Nepresolin or Nepressol or Ophthazin or Tonolysin). tw. (470)

249. 484-23-1. rn. (387)

250. exp Minoxidil/ (1306)

251. (Minoxidil or Alopexil or Alostil or Loniten or Lonolox or Mintop or Normoxidil or Prexidil or Regaine or Rogaine or Theroxidil or Tricoxidil). tw. (1378)

252. 38304-91-5. rn. (1306)

253. (Aliskiren or Tekturna or Enviage or Rasilez or Riprazo or Sprimeo). tw. (622)

254. Aliskiren. rn. (526)

255. exp Renin/ai [antagonists & inhibitors] (1585)

256. Clonidine/ (12516)

257. (Clonidine or Adesipress or Catapres$3 or (Catapres adj TTS*) or Chlophazolin or Clofelin or Clofenil or Clopheline or Dixarit or Duraclon or Gemiton or Hemiton or Isoglaucon or Klofelin or Klofenil or “Nexiclon XR”). tw. (13508)

258. exp Antihypertensive Agents/ (220309)

259. (antihypertensive* or anti-hypertensive*). tw. (37546)

260. (minizide or polypress). tw. (6)

261. (Polythiazide adj3 Prazosin). tw. (13)

262. 84057-89-6. rn. (2)

263. (Enduronyl or Enduron-deserpidine). tw. (1)

264. (Deserpidine adj3 methyclothiazide). tw. (7)

265. 8057-21-4. rn. (0)

266. “Diutensen-R”. tw. (0)

267. (Reserpine adj3 methyclothiazide). tw. (2)

268. or/8-267 (400861)

269. 7 and 268 (112506)

270. limit 269 to systematic reviews (2552)

271. meta analysis.pt. (37688)

272. exp meta-analysis as topic/ (12567)

273. (meta-analy* or metanaly* or metaanaly* or met analy* or integrative research or integrative review* or integrative overview* or research integration or research overview* or collaborative review*). tw. (49866)

274. (systematic review* or systematic overview* or evidence-based review* or evidence-based overview* or (evidence adj3 (review* or overview*)) or meta-review* or meta-overview* or “review of reviews” or technology assessment* or HTA or HTAs). tw. (64768)

275. exp Technology assessment, biomedical/ (8940)

276. health technology assessment winchester england.jn. (1031)

277. (evidence report technology assessment or evidence report technology assessment summary).j n. (204)

278. “cochrane database of systematic reviews”.jn. (9287)

279. or/271-278 (127337)

280. 269 and 279 (2137)

281. 270 or 280 (3526)

282. limit 281 to human (3414)

283. (in process or publisher or pubmed-not-medline or in-data-review).st. (1409788)

284. 281 and 283 (85)

285. 282 or 284 (3499)

286. (comment or editorial or interview or letter or news). pt. (1384444)

287. 285 not 286 (3359)

### **Appendix: Cochrane Database search strategy**

Database: EBM Reviews - Cochrane Database of Systematic Reviews <2005 to October 2012> Search Strategy:

1. Hypertension.kw. (157)

2. hypertens*.ti,ab,kw. (220)

3. ((high* or rais* or elevat* or heighten* or increas*) adj3 (“blood pressure” or “diastolic pressure” or “systolic pressure” or “pulse pressure”)).ti,ab,kw. (53)

4. ((high* or rais* or elevat* or heighten* or increas*) adj3 (BP or DBP or SBP)).ti,ab,kw. (10)

5. Cardiovascular Diseases pc.kw. (6)

6. ((borderline or pre-disease* or pre-clinical* or preclinical* or sub-clinical* or subclinical* or pre-morbid* or premorbid* or risk* or susceptib* or pre-dispos* or predispos* or predict* or probabilit* or likelihood or likeliness or prevent*) adj3 (cardiovascular or cardiometabolic* or cardio-metabolic* or coronary disease* or heart disease* or heart attack* or heart failure or myocardial infarction* or coronary artery disease* or CVD or peripheral artery disease* or PAD or CHD or CAD or arteriosclerosis or atherosclerosis or stroke)).ti,ab,kw. (267)

7. or/1-6 (470)

8. hypertension dt.kw. (17)

9. Sodium Chloride Symporter Inhibitors.kw. (3)

10. ((thiazide or benzothiadiazine or benzo-thiadiazine or potassium depleting) adj1 diuretic$1).ti,ab,kw. (10)

11. (sodium chloride symporter inhibitor$1 or sodium chloride cotransporter inhibitor$1 or sodium chloride co-transporter inhibitor$1 or thiazide sensitive NaCl cotransporter inhibitor$1 or thiazide sensitive NaCl co-transporter inhibitor$1).ti,ab,kw. (3)

12. Chlorothiazide.kw. (1)

13. (chlorothiazide or Alurene or Chlorosal or Chlotride or Chlorothiazid or Chlorothiazidum or Chlorthiazid or Chlorthiazide or Chlorthiazidum or Chlortiazid or Chlorurit or Chlotride or Clorotiazide or Clotride or Diuresal or Diuril or Diurilix or Diurite or Diutrid or Flumen or Minzil or Neo-dema or SK-Chlorothiazide or Salisan or Salunil or Saluretil or Saluric or Thiazide or Urinex or Warduzide or Yadalan).ti,ab,kw. (14)

14. Chlorthalidone.kw. (0)

15. (chlorthalidone or Apo-Chlorthalidone or Chlorphthalidolone or Chlorphthalidone or Chlortalidone or Chlortalidonum or Chlorthalidon or Clortalidone or Famolin or Hydro-Long or Hygroton or Igroton or Isoren or Natriuran or Oksodolin or oxodolin or Oradil or Oxodolin or Phthalamodine or Phthalamudine or Racemic chlorthalidone or Renon or Saluretin or Thalitone or Urolin or Zambesil).ti,ab,kw. (0)

16. Hydrochlorothiazide.kw. (0)

17. (Hydrochlorothiazide or “Aquazide H” or Apo-Hydro or Carozide or Dichlothiazide or Dihydrochlorothiazide or Esidrex or Esidrix or Ezide or HCTZ or Hydrochlorot or HydroDIURIL or Hydro-par or HydroSaluric or Hypothiazide or Microzide or Oretic or Sectrazide).ti,ab,kw. (1)

18. Hydroflumethiazide.kw. (0)

19. (Hydroflumethiazide or Bristab or Bristurin or Di-ademil or Di-adenil or Dihydroflumethiazide or Diucardin or Diuredemina or Diurometon or Elodrin or Elodrine or Enjit or Finuret or Flutizide or Hydol Hydrenox or Hydroflumethiazide or Hydroflumethiazidum or Hydroflumethizide or Idroflumetiazide or Leodrine or NaClex or Olmagran or Rivosil or Robezon or Rodiuran or Rontyl or saluron or Sisuril or Spandiuril or Trifluoromethylhydrothiazide or Vergonil).ti,ab,kw. (0)

20. Indapamide.kw. (0)

21. (indapamide or Arifon or Bajaten or Cormil or Damide or Fludex or Indaflex or Indapamide or Indamol or Ipamix or Lozol or Metindamide or Natrilix or Noranat or Pressurai or Tandix or Tertensif or Veroxil).ti,ab,kw. (0)

22. Methyclothiazide.kw. (0)

23. (methyclothiazide or Aquatensen or Enduron or Naturon).ti,ab,kw. (0)

24. Metolazone.kw. (0)

25. (metolazone or Diulo or Microx or Mykrox or Oldren or Zaroxolyn or Zytanix).ti,ab,kw. (0)

26. Polythiazide.kw. (0)

27. (Polythiazide or Drenusil or Nephril or Polythiazidum or Renese).ti,ab,kw. (0)

28. Angiotensin-Converting Enzyme Inhibitors.kw. (12)

29. ((Angiotensin-Converting Enzyme or Angiotensin I-Converting Enzyme or ACE or Kininase II) adj (inhibitor* or antagonist*)).ti,ab,kw. (45)

30. (ACEI or ACEIs).ti,ab,kw. (18)

31. Captopril.kw. (0)

32. (Acediur or Aceplus or Acepress or Acepril or Alopresin or Asisten or Capoten or Captolane or Captopril or Captoprilum or Captopryl or Captoril or Cesplon or Dilabar or Farcopril or Garranil or Hypertil or Hypopress or Isopresol or “L-Captopril” or Lopirin or Lopril or Novocaptopril or Tenosbon or Tensoprel or Zapto).ti,ab,kw. (2)

33. Enalapril.kw. (1)

34. (Enalapril or Bonuten or Enalaprila or Enalaprilum or Gadopril or Kinfil).ti,ab,kw. (2)

35. Lisinopril.kw. (0)

36. (Lisinopril or Lisinopril dehydrate or Prinivil or Renacor or Zestril).ti,ab,kw. (1)

37. (Benazepril hydrochloride or Benazepril HCl or Briem or Cibace or Cibacen or Cibacen CHF or Cibacene or Labopol or Lotensin or Lotrel or Tensanil or Zinadril).ti,ab,kw. (0)

38. Fosinopril.kw. (1)

39. (Fosinopril or Dynacil or Fosenopril or Fosinil or Fosinorm or Fositens or Fozitec or Hiperlex or Monopril or Newace or Staril or “Tenso Stop” or Tensocardil).ti,ab,kw. (1)

40. Ramipril.kw. (0)

41. (Ramipril or Acovil or Altace or Carasel or Cardace or Delix or Hytren or Lostapres or Naprix or Pramace or Quark or Ramace or Ramiprilum or Ramipro or Triatec or Tritace or Vesdil or Zabien).ti,ab,kw. (2)

42. (Quinapril hydrochloride or Accupril or Accuprin or Accupron or Acequin or Acuitel or Acuprel or Asig or Conan or Continucor or Ectren or Hemokvin or Korec or Koretic or Lidaltrin or Quinapril or Quinapril HCl or Quinazil).ti,ab,kw. (0)

43. Perindopril.kw. (1)

44. (Aceon or Covapril or Coversyl or Perindopril or Pirindopril or Prestarium).ti,ab,kw. (1)

45. (Trandolapril or Gopten or Mavik or Odrik or Udrik).ti,ab,kw. (0)

46. (Moexiril or Fempress or Moex or Moexipril hydrochloride or Perdix or Univasc).ti,ab,kw. (0)

47. Calcium Channel Blockers.kw. (35)

48. ((calcium or ca) adj2 (blocker* or blockader* or blocking or antagonist* or inhibitor*)).ti,ab,kw. (60)

49. Amlodipine.kw. (0)

50. (Amlodipine or Amlodipine Besylate or Amlodipine Maleate or Amlodis or Amlor or Astudal or Coroval or Istin or Lipinox or Norvasc).ti,ab,kw. (2)

51. (Aranidipine or Sapresta).ti,ab,kw. (0)

52. (Azelnidipine or Calblock).ti,ab,kw. (0)

53. (Barnidipine or Cyress or HypoCa or Libradin or Mepirodipine).ti,ab,kw. (0)

54. (Benidipine or Benidipinum or Coniel).ti,ab,kw. (0)

55. (Cilnidipine or Atelec or Cinalong or Siscard).ti,ab,kw. (0)

56. (Clevidipine or Cleviprex).ti,ab,kw. (0)

57. Isradipine.kw. (0)

58. (Isradipine or Dynacirc or “DynaCirc CR” or Isradipinum or Lomir or Prescal).ti,ab,kw. (1)

59. (Efonidipine or Landel).ti,ab,kw. (0)

60. Felodipine.kw. (0)

61. (Felodipine or Agon or Felo Biochemie or Felo-Puren or Felobeta or Felocor or Felodipin or Felodur or Felogamma or Fensel or Flodil or Modip or Munobal or Perfudal or Plendil or Renedil).ti,ab,kw. (0)

62. (Lacidipine or Lacidipinum or Lacimen or Lacipil or Motens).ti,ab,kw. (0)

63. (Lercanidipine or Lacidipinum or Lercadip or Lerdip or Zanidip).ti,ab,kw. (0)

64. (Manidipine or Calslot or Madipine or Franidipine).ti,ab,kw. (0)

65. Nicardipine.kw. (0)

66. (Nicardipine or Antagonil or Carden SR or Cardene or Dagan or Flusemide or Lecibral or Lincil or Loxen or Lucenfal or Nicardipinum or Perdipine or Ridene or Vasonase).ti,ab,kw. (1)

67. Nifedipine.kw. (5)

68. (Nifedipine or Adalat or Afeditab or Citilat or Cordipin or Cordipine or Corinfar or Fenihidin or Fenihidine or Fenigidin or Korinfar or Nifediac or Nifedical or Nifangin or Oxcord or Procardia or Procardia XL or Vascard).ti,ab,kw. (10)

69. (Nilvadipine or Escor or Nivadil or Nilvadipinum).ti,ab,kw. (0)

70. Nimodipine.kw. (5)

71. (Nimodipine or Admon or Brainal or Calnit or Kenesil or Modus or Nimodipin or Nimodipinum or Nimotop or Periplum or Remontal).ti,ab,kw. (10)

72. Nisoldipine.kw. (0)

73. (Nisoldipine or Baymycard or Nisocor or Nisoldipinum or Sular or Syscor).ti,ab,kw. (0)

74. Nitrendipine.kw. (0)

75. (Nitrendipine or Balminil or Bayotensin or Baylotensin or Baypresol or Baypress or Cardif or Gericin or Jutapress or Nidrel or Niprina or Nitre AbZ or Nitre-Puren or Nitregamma or Nitren 1A Pharma or Nitren acis or Nitren Lich or Nitrend KSK or Nitrendepat or Nitrendi Biochemie or Nitrendidoc or Nitrendimerck or Nitrepin or Nitrendipin or Nitrendipino or Nitrensal or Nitrepress or Nitrendipinum or Tensogradal or Trendinol or Vastensium).ti,ab,kw. (0)

76. (Pranidipine or Acalas).ti,ab,kw. (0)

77. Verapamil.kw. (3)

78. (Verapamil or Calan or Cordilox or Dexverapamil or Dilacoran or Falicard or Finoptin or Iproveratril or Isoptimo or Isoptin or Isoptine or Izoptin or Lekoptin or Vasolan or Verapamilum or dl-Verapamil).ti,ab,kw. (6)

79. Diltiazem.kw. (1)

80. (Diltiazem or Aldizem or Cardil or Cardizem or Cardizem LA or Dilacor or Dilacor XR or Dilcontin or Dilren or Dilta-Hexal or Diltiazem Hydrochloride or Diltiazem Malate or Diltiazemum or Dilticard or Dilzem or Endrydil or Incoril AP or Tiazac).ti,ab,kw. (3)

81. Mibefradil.kw. (0)

82. (mibefradil or Posicor).ti,ab,kw. (1)

83. Bepridil.kw. (0)

84. (Bepridil or Bedapin or Bepadin or Cordium or Unicordium or Vascor).ti,ab,kw. (0)

85. Fluspirilene.kw. (1)

86. (fluspirilene or Fluspi or Fluspirilenum or Imap or Kivat or Redeptin).ti,ab,kw. (1)

87. Aldosterone Antagonists.kw. (3)

88. (aldosterone adj (antagonist* or inhibit*)).ti,ab,kw. (6)

89. Spironolactone.kw. (2)

90. (spironolactone or Acelat or Aldace or Aldactone or Alderon or Aldopur or Almatol or Altex or Aquareduct or Berlactone or duraspiron or Diatense or Espironolactona or Euteberol or Flumach or Frumikal or Jenaspiron or Novo-Spiroton or Practon or Spiractin or Spiresis or Spirobeta or Spirogamma or Spirolactone or Spirolang or Spirono-Isis or Spironone or Spirospare or Uractone or Urusonin or Veroshpiron or Verospiron or Verospirone or Xenalon).ti,ab,kw. (3)

91. (Eplerenone or Inspra).ti,ab,kw. (1)

92. Adrenergic Antagonists.kw. (1)

93. ((Adrenergic or alpha-adrenergic or beta-adrenergic) adj3 (block* or alpha-block* or beta-block* or antagonist* or alpha-antagonist* or beta-antagonist*)).ti,ab,kw. (47)

94. ((alpha1 or “alpha-1” or alpha2 or “alpha-2” or beta or beta1 or “beta-1” or beta2 or “beta-2” or beta3 or “beta-3”) adj2 (block* or antagonist*)).ti,ab,kw. (69)

95. (adrenolytic* or anti-adrenergic* or antiadrenergic*).ti,ab,kw. (0)

96. Phenoxybenzamine.kw. (0)

97. (Phenoxybenzamine or Bensylyt or Benzylyt or Dibenylene or Dibenyline or Dibenziran or Dibenzylin or Dibenzyline or Dibenzyran or Fenossibenzamina or Fenoxibenzamina or Phenoxybenzaminum).ti,ab,kw. (0)

98. Phentolamine.kw. (0)

99. (Phentolamine or Dibasin or Fentolamin or Phentolaminum or Regitine or Regityn or Rogitine or “Z-Max”).ti,ab,kw. (0)

100. Tolazoline.kw. (0)

101. (Tolazoline or Artonil or Benzalolin or Benzazoline or Benzidazol or Benzolin or Benzylimidazoline or Dilatol ASI or Divascol or Imidalin or Kasimid or Lambril or Olitensol or Peripherine or Phenylmethylimidazoline or Prefaxil or Pridazole or Priscol or Priscoline or Tolazolin or Tolazolinum or Vasimid or Vasodil or Vasodilatan).ti,ab,kw. (0)

102. (Alfuzosin or Alfetim or Afusozine or Alphuzosine or Alfuzosinum or Benestan or Urion or UroXatral or Xatral).ti,ab,kw. (2)

103. Prazosin.kw. (2)

104. (Prazosin or Furazosin or Minipress or Pratsiol or Prazosinum).ti,ab,kw. (2)

105. Doxazosin.kw. (2)

106. (Doxazosin or Alfamedin or Apo-Doxazosin or Cardular or Cardura or Carduran or Carduran Neo or Diblocin or Doxa-Puren or Doxacor or Doxagamma or Doxamax or Doxatensa or DoxaUro or Doxazomerck or Doxazosine or Doxazosinum or Gen-Doxazosin or Jutalar or MTW-Doxazosin or Novo-Doxazosin or Progandol Neo or Uriduct or Zoxan).ti,ab,kw. (3)

107. (Tamsulosin or Flomax or Tamsulosine or Tamsulosinum).ti,ab,kw. (5)

108. (Terazosin or Adecur or Apo-Terazosin or Blavin or Deflox or Dysalfa or Flotrin or Flumarc or Fosfomic or Heitrin or Hytrin or Hytrine or Magnurol or Novo-Terazosin or Nu-Terazosin or Sutif or Tazusin or Terazoflo or Vasomet or Zayasel).ti,ab,kw. (3)

109. (Atipamezole or Antisedan or Atipamezol or Atipamezolum).ti,ab,kw. (0)

110. Idazoxan.kw. (0)

111. (Idazoxan or Idazoxanum).ti,ab,kw. (0)

112. Yohimbine.kw. (1)

113. (Yohimbine or Aphrosol or Aphrodine or Aphrodyne or Corynine or Corynanthine or Pluriviron or Quebrachin or Quebrachine or Rauhimbine or Rauwolscine or Yocon or Yohimbin or Yohimex).ti,ab,kw. (2)

114. (Carvedilol or Carvedilolum or Coreg or Coropres or Dilatrend or Eucardic or Kredex or Querto).ti,ab,kw. (1)

115. Labetalol.kw. (0)

116. (Labetalol or Albetol or Apo-Labetalol or Dilevalol or Dilevalolum or Labetolol or Normodyne or Presolol or Trandate).ti,ab,kw. (1)

117. Alprenolol.kw. (0)

118. (Alprenolol or Alfeprol or Alpheprol or Alprenololum or Aptin or Aptin-Duriles or Aptina or Aptine).ti,ab,kw. (0)

119. (Bucindolol or Bucindololum).ti,ab,kw. (0)

120. Carteolol.kw. (0)

121. (Carteolol or Carteololum).ti,ab,kw. (0)

122. Nadolol.kw. (0)

123. (Nadolol or Anabet or Corgard or Corzide or Nadololum or Solgol).ti,ab,kw. (0)

124. Oxprenolol.kw. (0)

125. (Oxprenolol or Coretal or Koretal or Oxprenololum or Slow Trasicor or Tevacor or Trasicor).ti,ab,kw. (1)

126. Penbutolol.kw. (0)

127. (Penbutolol or Betapressin).ti,ab,kw. (0)

128. Pindolol.kw. (2)

129. (Pindolol or Betapindol or “Blocklin L” or Calvisken or Carvisken or Decreten or Durapindol or Glauco-Viskin or Pectobloc or Pinbetol or Pindololum or Prinodolol or Pynastin or Visken).ti,ab,kw. (2)

130. Propranolol. kw. (5)

131. (Propranolol or Anaprilin or Anapriline or Avlocardyl or Betadren or Betalong or beta-Propranolol or Corpendol or Dexpropranolol or Dociton or Euprovasin or Inderal or Obsidan or Obzidan or Propanix or Propranololum or Reducor or Sawatal or Sumial or Rexigen).ti,ab,kw. (9)

132. Sotalol.kw. (0)

133. (Sotalol or Darob or Sotalolum or beta-Cardone).ti,ab,kw. (2)

134. Timolol.kw. (1)

135. (Timolol or Blocadren or Timacar).ti,ab,kw. (3)

136. Eucommia bark$1.ti,ab,kw. (0)

137. Atenolol.kw. (1)

138. (Atenolol or Tenormin or Tenormine).ti,ab,kw. (2)

139. Betaxolol.kw. (0)

140. (Betaxolol or Betaxololum).ti,ab,kw. (0)

141. Bisoprolol.kw. (0)

142. (Bisoprolol or Concor).ti,ab,kw. (0)

143. Celiprolol.kw. (1)

144. (Celiprolol or Celiprololum or Selectol).ti,ab,kw. (1)

145. Esmolol.ti,ab,kw. (0)

146. Metoprolol.kw. (1)

147. (Metoprolol or Beatrolol or Beloc-Duriles or Betaloc or Betalok or Corvitol or Lopressor or Meijoprolol or Metohexal or Metoprololum or Metrol or Minax or Neobloc or Preblok or Presolol or Selokeen or Seloken or Spesicor or Spesikor or Toprol).ti,ab,kw. (2)

148. (Nebivolol or Bystolic or Lobivon or Nebilet or Nobiten or Silostar or Vasoxen).ti,ab,kw. (0)

149. Angiotensin Receptor Antagonists.kw. (7)

150. (angiotensin adj3 (antagonist* or block*)).ti,ab,kw. (18)

151. (Sartan or Sartans).ti,ab,kw. (0)

152. ARBS.ti,ab,kw. (9)

153. Losartan.kw. (1)

154. (Losartan or Cozaar or Losartan Monopotassium Salt or Losartan Potassium).ti,ab,kw. (1)

155. Candesartan.ti,ab,kw. (0)

156. (Valsartan or Diovan or Kalpress or Miten or Nisis or Provas or Tareg or Vals or Valtan or Valzaar).ti,ab,kw. (0)

157. (Irbesartan or Aprovel or Avapro or Karvea).ti,ab,kw. (0)

158. (Telmisartan or Kinzalmono or Micardis or Pritor).ti,ab,kw. (0)

159. (Eprosartan or Teveten).ti,ab,kw. (0)

160. 160 (Benicar or Olmesartan or Omesartan or Olmetec or Votum).ti,ab,kw. (0)

161. Azilsartan.ti,ab,kw. (0)

162. Hydralazine.kw. (1)

163. (Hydralazine or Apresoline or Apressin or Apressoline or Aprezolin or Hydrallazin or Hydrazinophthalazine or Nepresol).ti,ab,kw. (5)

164. (Dihydralazine or Depressan or Dihydrallazine or Dihydrazinophthalazin or Hypopresol or Nepresol or Nepresolin or Nepressol or Ophthazin or Tonolysin).ti,ab,kw. (0)

165. Minoxidil.kw. (3)

166. (Minoxidil or Alopexil or Alostil or Loniten or Lonolox or Mintop or Normoxidil or Prexidil or Regaine or Rogaine or Theroxidil or Tricoxidil).ti,ab,kw. (3)

167. (Aliskiren or Tekturna or Enviage or Rasilez or Riprazo or Sprimeo).ti,ab,kw. (1)

168. Renin ai.kw. (0)

169. Clonidine.kw. (8)

170. (Clonidine or Adesipress or Catapres$3 or (Catapres adj TTS*) or Chlophazolin or Clofelin or Clofenil or Clopheline or Dixarit or Duraclon or Gemiton or Hemiton or Isoglaucon or Klofelin or Klofenil or “Nexiclon XR”).ti,ab,kw. (18)

171. Antihypertensive Agents.kw. (46)

172. (antihypertensive* or anti-hypertensive*).ti,ab,kw. (77)

173. (minizide or polypress).ti,ab,kw. (0)

174. (Polythiazide adj3 Prazosin).ti,ab,kw. (0)

175. (Enduronyl or Enduron-deserpidine).ti,ab,kw. (0)

176. (Deserpidine adj3 methyclothiazide).ti,ab,kw. (0)

177. “Diutensen-R”.ti,ab,kw. (0)

178. (Reserpine adj3 methyclothiazide).ti,ab,kw. (0)

179. or/8-178 (240)

180. 7 and 179 (108)

## Abbreviations

ACE: Angiotensin-converting enzyme; ARB: Angiotensin receptor blockers; BP: Blood pressure; CHEP: Canadian Hypertension Education Program; DBP: Diastolic blood pressure; DSR: Distiller Systematic Review Software; MI: Miacardial infarction; NMA: Network meta-analysis; PRESS: Peer Review of Electronic Search Strategies; RCTs: Randomized controlled trials; SBP: Systolic blood pressure.

## Competing interests

DF, LB, DM, SK, SS, AT, JT, FY and JT have no competing interests to declare. BH has received speakers’ fees from Amgen Canada in relation to issues regarding network meta-analysis. EM has consulted with Merck & Co. Inc., Pfizer Ltd., Novartis, Takeda and GlaxoSmith Kline on network meta-analysis issues and receives salary support from the Canadian Institutes of Health Research through a Canada Research Chair. KT has consulted with Merck & Co. Inc., Pfizer Ltd., Novartis, Takeda and GlaxoSmith Kline on network meta-analysis issues. FHHL holds the Pfizer Chair in Hypertension Research, an endowed chair supported by Pfizer Canada, the University of Ottawa Heart Institute Foundation and the Canadian Institutes of Health Research.

## Authors’ contributions

BH, DM, DF, SK and FL contributed to the design of the project plan and the corresponding methods chosen. BH, DM and SK were responsible for preparation of the original protocol draft. FL provided clinical expertise throughout the protocol development process. JT, FY and JT are involved in the conduct of this work. All authors provided critical review of the manuscript and the corresponding peer reviewed protocol that was submitted to and successfully funded by the Canadian Institutes of Health Research and the Drug Safety and Effectiveness Network. All authors read and approved the final manuscript.
